# Neuroimaging-derived brain-age: an ageing biomarker?

**DOI:** 10.18632/aging.101286

**Published:** 2017-08-30

**Authors:** James H. Cole

**Affiliations:** Computational, Cognitive and Clinical Neuroimaging Laboratory, Imperial College, London, UK

**Keywords:** neuroimaging, ageing biomarker, brain volume, biomarker criteria

The search for robust, reliable and valid biomarkers of the ageing process is a key goal for gerontological science. Such tools should enable the quantification of individual differences in underlying biological ageing. This could have great utility for mapping personalised ageing trajectories, for predicting risk of future age-related deterioration and disease and for evaluating potential treatments aimed at improving healthspan or even slowing ageing itself. Given the multi-faceted nature of biological ageing, numerous potential candidate biomarkers have been proposed. These can be anthropometric, physiological or blood-based; indexing immune function, epigenetic signatures, gene expression profiles, physical capacity or body composition [1]. To improve on individual predictors of biological age, panels combining multiple markers have also been proposed [2]. While many of these approaches are highly promising, the results have yet to be translated into clinical practice.

The criteria most commonly used for assessing the appropriateness of ageing biomarkers is how strongly they correlate with chronological age in healthy people. In addition, thanks to the increasing use of machine learning, the accuracy with which chronological age can be predicted using multivariate biological data is also a useful indicator of potential biomarker value. Aligned with this, an independent line of research has emerged from the field of neuroscience. Using neuroimaging data, principally magnetic resonance imaging (MRI) brain scans, chronological age can be predicted accurately in a machine-learning framework [3]. This *neuroimaging-derived brain-age* model is based on data from over 2000 healthy adults and shows excellent test-retest reliability. This presents the intriguing possibility that *in-vivo* measurements of brain volume could be used as an alternative ageing biomarker.

It is well-known that ageing affects the brain, both in terms of outward behavioural changes and cognitive decline, alongside alterations to the brain's biophysical structure and cellular and molecular functioning. Using measures of brain volume derived from T1-weighted structural MRI, assumed to reflect grey and white matter atrophy, high levels of age prediction accuracy have been consistently achieved. For example, our work found a mean/median absolute error of age prediction of 4.2/3.4 years, with a correlation between age and brain-predicted age of *r* = 0.96, R^2^ = 0.92 [3]. This is comparable to or better than leading biological age prediction models, for example using DNA methylation status (*r* = 0.96, median absolute error = 3.6 years) [4] or a panel of blood chemistry markers (*r* = 0.91, mean absolute error = 5.6 years) [2].

Given the excellent performance of neuroimaging-derived age predictions, it is curious how brain volume has not been considered more widely as a candidate ageing biomarker. For example, neuroimaging is not mentioned in the recent review by Wagner and colleagues [1] nor in a collaboratively-developed proposed panel of ageing biomarkers [5]. Our data suggest that the addition of neuroimaging to the pantheon of candidate ageing biomarkers could be highly beneficial. When predicting time to mortality, arguably the ‘gold standard’ of health outcomes, we showed that neuroimaging-derived brain-age outperformed both DNA-methylation age and leukocyte telomere length [6]. In fact, telomere length was only related to survival at chance levels. Interestingly, when neuroimaging-derived brain-age and DNA-methylation age, themselves uncorrelated, were combined in statistical model to predict survival in older adults, the resulting model significantly outperformed a model containing each predictor individually. This suggests that biological ageing can potentially be compartmentalised, and that when premature ageing occurs in different compartments (e.g., haemo-epigenetic, neurological), the risk of poor health outcomes is substantially increased.

Given the published data on neuroimaging-derived brain-age, I believe it is worth considering it qualification against a set of consensus ageing biomarker criteria. Paraphrasing from the American Federation for Aging Research recommendations, an ageing biomarker must:

1. Predict the rate of ageing (i.e., estimate where a person is in their total life span).
2. Measure a basic process that underlies ageing, not the effects of disease.
3. Be able to be tested repeatedly without causing harm.
4. Work in humans and laboratory animals.

Based on the above evidence regarding prediction of survival [6], I assert that neuroimaging-derived brain-age meets criteria #1. Given the accuracy of age prediction [3] and the fact that brain atrophy occurs in the context of non-pathological ageing, this satisfies criteria #2. As a non-invasive imaging technique, T1-weighted MRI meets criteria #3. Finally, the accuracy of this technique in non-human primates has been recently reported [7], suggesting that it appropriately meets criteria #4. While perhaps the major caveat regarding the use of neuroimaging in this context is the cost and potential logistics, projects like the UK Biobank imaging study (http://imaging.ukbiobank.ac.uk/) show that collecting neuroimaging data on an extremely large scale (N = 100,000) are becoming increasingly feasible. It is timely for a marriage of neuroscience and biogerontology, and approaches that combine the most complementary information on the ageing human body will have the greatest utility in developing effective ageing biomarkers.
